# Prevention of traumatic corneal ulcer in South East Asia

**Published:** 2017

**Authors:** M. Srinivasan

**Affiliations:** Director Emeritus, Aravind Eye Care, Madurai, Tamil Nadu India.

**Figure:**
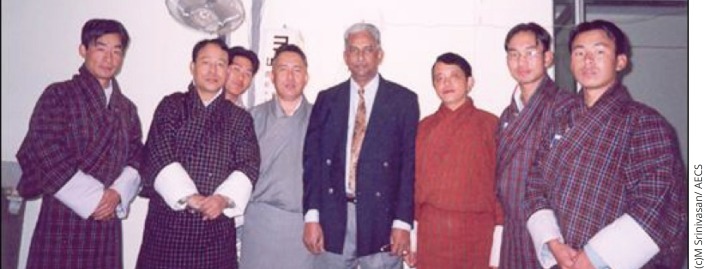
Country principal investigator and lead principal investigator with village health workers in Bhutan

**Figure:**
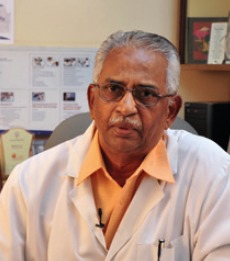
Dr. M. Srinivasan

## Introduction

Corneal ulceration is a leading cause of visual impairment globally, with a disproportionate burden in developing countries. It was estimated that 6 million corneal ulcers occur annually in the ten countries of South East Asia region encompassing a total population of 1.6 billion.^1^ While antimicrobial treatment is generally effective in treating infection, “successful” treatment is often associated with a poor visual outcome.

The scarring that accompanies the resolution of infection leaves many eyes blind. Thus, prevention of corneal ulceration is important to reduce morbidity associated with corneal ulceration in countries grouped under South Asian Association for Regional Cooperation (SAARC). Traditional infectious causes of blindness, such as trachoma, onchocerciasis, and leprosy, are declining, and soon the majority of corneal blindness will be due to microbial keratitis. Most corneal ulcers occur among agricultural workers in developing countries following corneal abrasion.

Several non-randomised prevention studies conducted before 2000 (Bhaktapur Eye Study)^2^ and during 2002 to 2004 in India, Myanmar, and Bhutan by World Health Organization (WHO), have suggested that antibiotic ointment applied promptly after a corneal abrasion could lower the incidence of ulcers, relative to neighbouring or historic controls.^3–4^ Prevention of traumatic corneal ulcer adopting the Bhaktapur model in a multicountry study in India, Bhutan, Myanmar during 2002 to 2004 was sponsored by WHO.

## Methods

The manpower utilised for this multi country study to identify ocular injury and treat corneal abrasion is given below:

**Bhutan:** Volunteer Village Health Workers (VVHW) of the Government were utilised to identify ocular injury and treat corneal abrasion

**Myanmar:** Village Health Workers (VHW) of the health department

**India:** paid village volunteers were utilised

## Inclusion criteria

- Resident of study area
- Corneal abrasion after ocular injury, confirmed by clinical examination with fluorescein stain and a blue torch
- Reported within 48 hours of the injury
- Subject aged >5 years of age

**Table 1.** Study design, sample size and results

	Bhutan	India	Myanmar
**Study design**	Unmasked	Randomized double masked trial	Unmasked
**Sample size**	111 corneal abrasions	338 corneal abrasions	111 corneal abrasions
**Results of Multicountry study**			
**No. of ocular injuries**	135	1365	273
**No. of corneal abrasions**	115	409	126
**No. of eligible corneal abrasions**	115	374	126
**Adverse events**	Nil	4	Nil

## Exclusion criteria

- Subject not a resident of study area
- Presence of clinically evident corneal infection
- Penetrating corneal injury or stromal laceration
- Bilateral ocular trauma

The study was approved by Institutional Review Boards (IRB) from all the three countries.

## Treatment Protocol

In general, corneal abrasions are treated with topical antibiotics and cycloplegics. In few centres bandaging the affected eye is practiced but it is controversial. 1% chloramphenicol ointment and 1% clotrimazole ointment was used. In Bhutan only chloramphenicol was used. In Myanmar both antibiotic and antifungal ointment applied but in India it was randomised and one arm was masked to receive both and in the other arm chloramphenicol and placebo ointment were used to find out whether antifungal prophylax is needed to prevent fungal ulcer. Frequency of application of all the drugs was three times a day for three days, supervised by village eye workers for compliance.

## Conclusion of multi-country study

**Figure 1.**
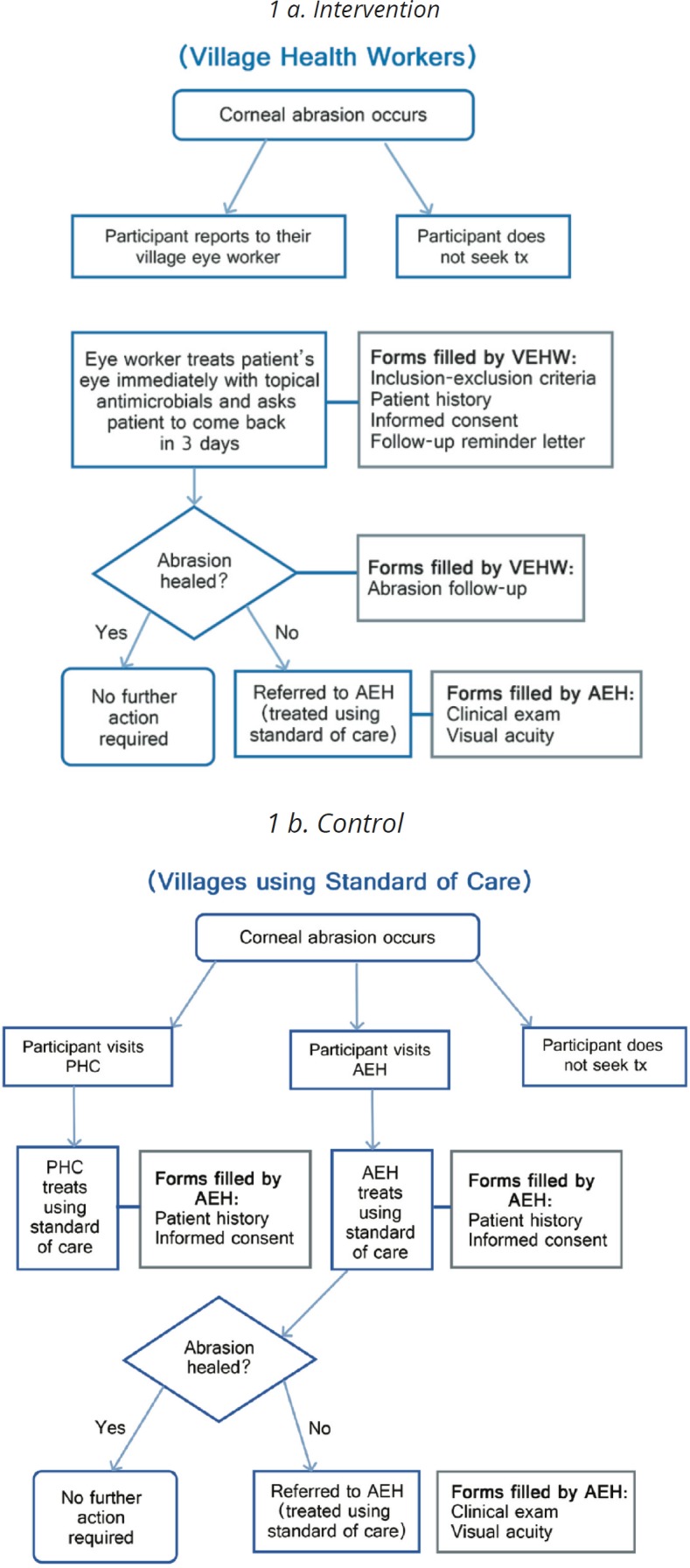
Design of the study for intervention and control arms

This model of delivering eye care services through trained village eye workers and grass root health workers is a replicable model for any developing country, especially for SAARC countries. (Figure 1a). Follow up rate on the third day at all centres were more than 98%. No case of serious adverse event was reported. Developing bacterial and fungal corneal ulcer using 1% chloramphenicol ointment in 96% of patients could be prevented if reported within 24 hours.

In Madurai, South India, a clinical trial during the same period demonstrated that abrasions randomised to topical antibacterial and antifungal prophylaxis were not significantly less likely to develop fungal ulcers than those randomised to antibacterial ointment alone, even though the region had a high incidence of fungal infection. This same trial also found that the incidence of ulcers in villages outside the prophylaxis programme one was far higher; these control villages were neighbouring, but not randomised. To address this issue, we proposed a community randomised trial comparing villages randomised to receive an intervention consisting of a trained village eye worker identifying, escorting or referring the patient from intervention villages to the nearest vision centre run by Aravind Eye Care System. There a trained vision technician would confirm corneal abrasion, provide 1% chloramphenical ointment to the eligible, enrolled patients in Madurai district. Control villages received no additional intervention. The primary outcome of corneal ulcer prevention will be measured by baseline and annual population-based census performed in both intervention and control villages by masked examiners from baseline to 24 months. The examiners will examine the eyes of all households from intervention and non-intervention villages who are suspected of having a corneal ulcer or injury with torch light and magnifying loup.

Each resident in the village will be examined for evidence of corneal opacity and asked about their ocular history.

Annual visits will occur in villages randomised to the intervention, an active promotion campaign will be undertaken to urge residents to notify the village eye health worker within 24 hours of ocular trauma. In control villages, abrasions and ulcers will be treated if they present to the vision centre or are found during annual monitoring visits, but active promotion of corneal abrasion care will not be offered.

## Methodology – phase II study

The study was designed in 2014 in consultation with F.I. Proctor Foundation, San Francisco. Enrollment began in January 2015 and ended in December 2016. 42 villages having approximately 92 thousand people involved in agriculture work were allotted. Randomisation was done to have 50% of the population for intervention (Figure 1b) and rest for non-intervention (Figure 1b). Data collection, entry, and analysis will be done at Aravind Eye Care System, Madurai and the project will be completed in 2017. Legal and ethical clearance was obtained from appropriate authorities. Details of the study design are in the flow chart. (Figure 1)

## Primary outcome of the current study

The primary outcome will be the incidence of corneal ulceration in the two study arms as measured by corneal examination at the base hospital or vision centre with telemedicine facility. In this study 1% chloramphenicol ointment and 1% itraconazole ointment is applied three times a day for three days and compliance would be checked by village health workers. Any adverse event will be informed to the study Principal Investigator (PI) or Aravind Eye Hospital and will be taken care at no cost to the participant.

## Manpower

20 paid village workers who have completed school and are able to fill study forms in English and reside in the study village were enrolled. Two supervisors well-experienced in rural eye care work will oversee the workers.

### Steps in managing programme

- Workers attend one week training at Aravind Eye Hospital prior to the study to learn basic anatomy of the eye, common corneal and external diseases, ocular injuries, vision testing, use of fluorescein strip, simple eye medicines application and; attend twice-yearly refresher training throughout the course of the study.
- Promote awareness of corneal abrasion intervention in intervention villages only
- Accessible by villagers via mobile phone
- Conduct eye examination to diagnose corneal abrasion and/or ulcer
- Assist treatment at vision centre for corneal abrasion, and follow up with the patient for three days at the village to ensure compliance
- Motivate patient to return for follow-up three days after treatment, assess compliance and perform examination
- If the patient develops a corneal ulcer or adverse reaction, he/she is referred to Aravind Eye Hospital for immediate treatment.

Verbal consent must be obtained from all individuals who present to the village eye worker to receive study medication. Possible risks and benefits of receiving the treatment will be explained. For patients under 18, both the child and one parent or guardian will provide consent for the child's participation.

We believe the results of this study may emerge as a replicable model to prevent traumatic corneal ulcer, and reduce corneal blindness in South Asia.
